# Supplementary material for: In situ observation of crystal rotation in Ni-based superalloy during additive manufacturing process
Source: Nat Commun. 2023 May 23;14:2961. doi: 10.1038/s41467-023-38727-8 (PMC10206100; doi:10.1038/s41467-023-38727-8)
Supplement: Supplementary file 1 — Supplementary Information [file 41467_2023_38727_MOESM1_ESM.pdf]

Supplementary Information

In situ observation of crystal rotation in Ni-based superalloy during additive manufacturing process

Dongsheng. Zhang[1,2,*], Wei. Liu[3,*], Yuxiao. Li[4], Darui. Sun[1], Yu. Wu[3], Shengnian. Luo[4], Sen. Chen[5,**], Ye. Tao[1,**], Bingbing. Zhang[1,2,**]

[1] Institute of High Energy Physics, Chinese Academy of Sciences, 19B Yuquan Road, Beijing, People's Republic of China

[2] University of Chinese Academy of Sciences, 19A Yuquan Road, Beijing, People's Republic of China

[3] 3D Printing Research and Engineering Technology Center, Beijing Institute of Aeronautical Materials, Beijing, People's Republic of China

[4] The Peac Institute of Multiscale Sciences, Chengdu, Sichuan, People's Republic of China

[5] Institute of Fluid Physics, China Academy of Engineering Physics, Mianyang, Sichuan 621900, China

[*]These authors contribute equally to this work.

[**]Corresponding authors

Email addresses: chensen@mail.ust.edu.cn(Sen. Chen), taoy@ihep.ac.cn (Ye. Tao), zhangbb@ihep.ac.cn (Bingbing. Zhang)

Supplementary Table 1. Chemical composition of the SX nickel-based superalloy (wt.%).

Co	Cr	Mo	W	Al	Ta	Hf	Re	Ni
7.5	7.0	1.5	5.0	6.2	6.5	0.15	3.0	Bal.

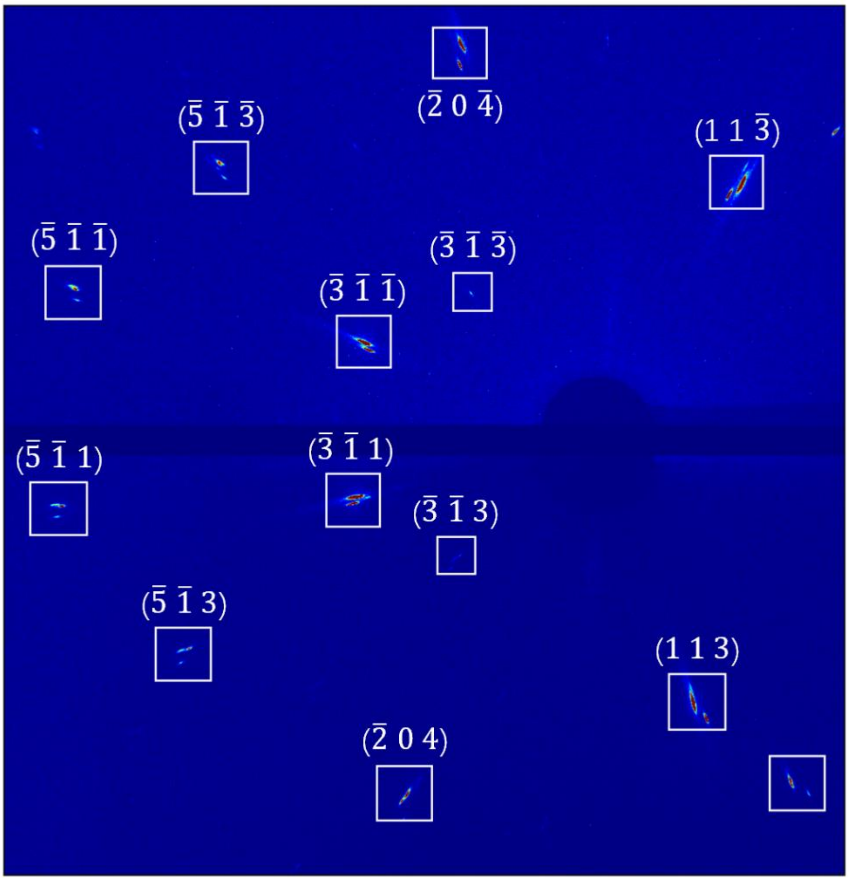

Supplementary Fig. 1. Laue diffraction pattern of the initial matrix metal recorded with exposure time of 50 ms. The Laue reflections are indexed in white.

Laue spot indexing is conducted by comparing the experimental results to the simulated Laue diffraction pattern generated by LauePt [1]. The diffracted spots correspond to the γ phase of the SX nickel-based superalloy.

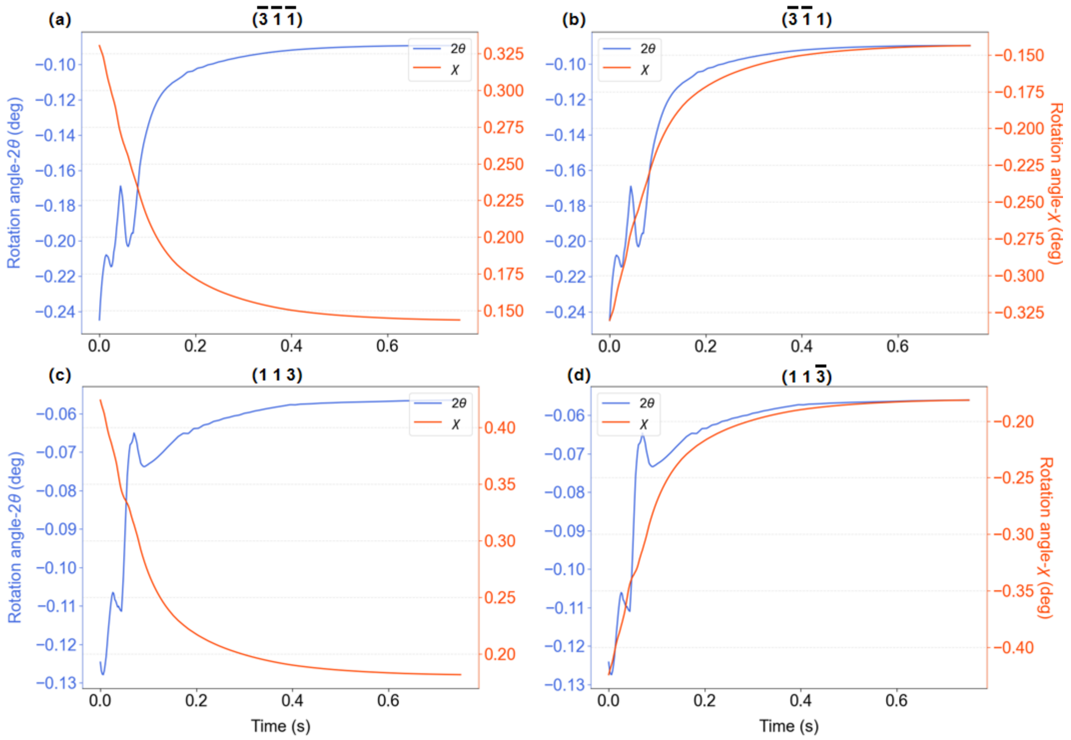

Supplementary Fig. 2. Laue diffraction simulated rotation angles of different lattice planes caused by the strain during solidification. Simulated rotation angles of the γ($\bar{3}\,\bar{1}\,\bar{1}$) (a), γ($\bar{3}\,\bar{1}\,1$) (b), γ($1\,1\,3$) (c) and γ($1\,1\,\bar{3}$) (d) lattice planes along the 2θ (blue lines) and χ (orange lines) directions.

We conduct a Laue diffraction simulation[2], to quantify the contribution of deformation to the Bragg peak shift (i.e., rotation of lattice planes), using the strain tensor (Fig. 5) obtained from the thermomechanical simulation as input. Supplementary Fig. 2 shows the angular shift caused by the strain effect along the 2θ and χ directions.

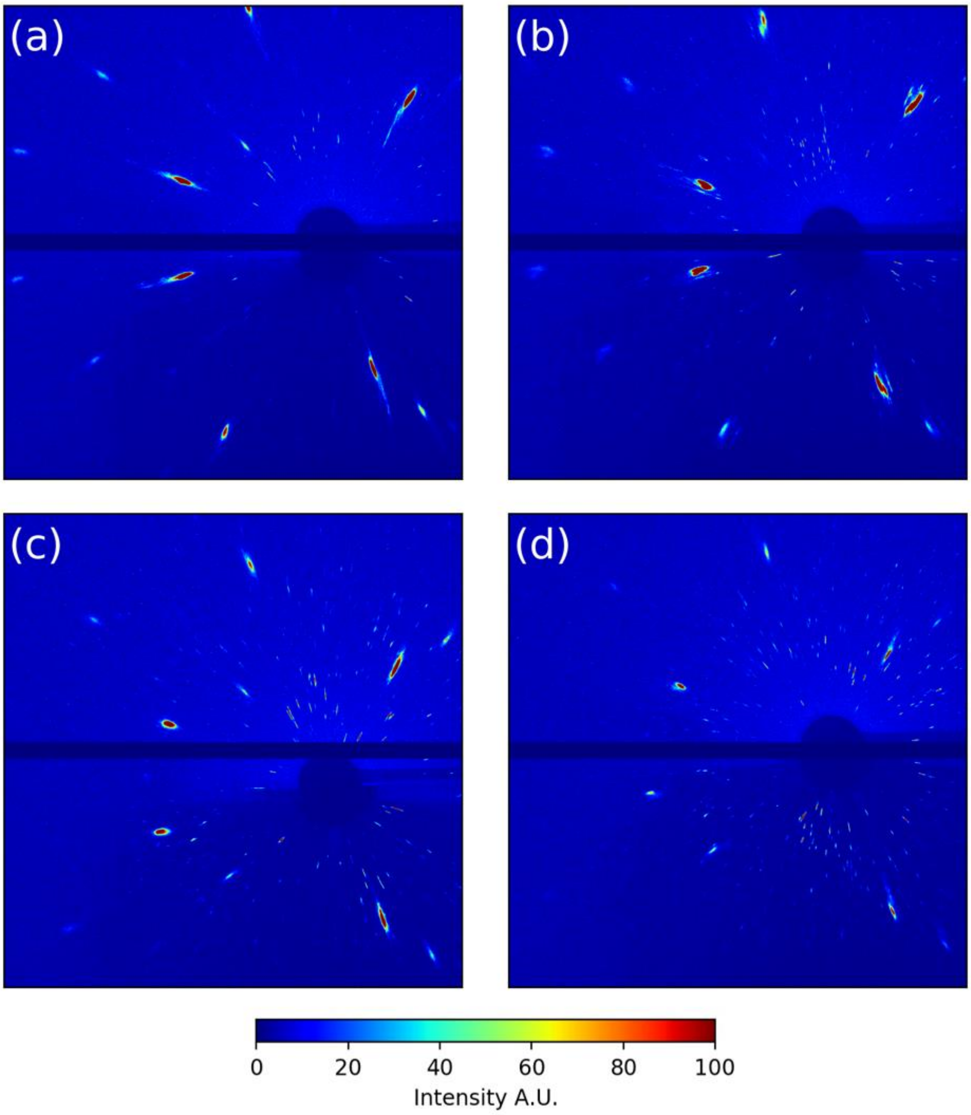

Supplementary Fig. 3. Final-state Laue diffraction patterns captured after solidification under different laser powers. Four laser powers are used: 245 W (a), 266 W (b), 350 W (c) and 385 W (d).

A higher content of SGs leads to the appearance of more sporadic diffraction spots because the SG orientation differs from that of the epitaxial grains. Therefore, the number of Laue diffraction spots can reflect the content of SGs to some extent. To determine the diffraction spot number, we set intensity and area thresholds for diffraction spots. To avoid a situation where a large fraction of SGs generates only a few diffraction spots due to the large size of the SGs, we set the intensity threshold to 0.15-times the strongest intensity of single-crystal diffraction spots, rather than using a constant threshold. This is because the intensity of single-crystal diffraction spots is proportional to the volume of the single crystal. When the intensity of single-crystal diffraction spots is low, the intensity threshold is also low, and more relatively lower-intensity diffraction spots can be chosen. To avoid errors caused by dead pixels and single-pixel statistical fluctuations, we use an area threshold of 4 pixels. The results are presented in Fig. 4b.

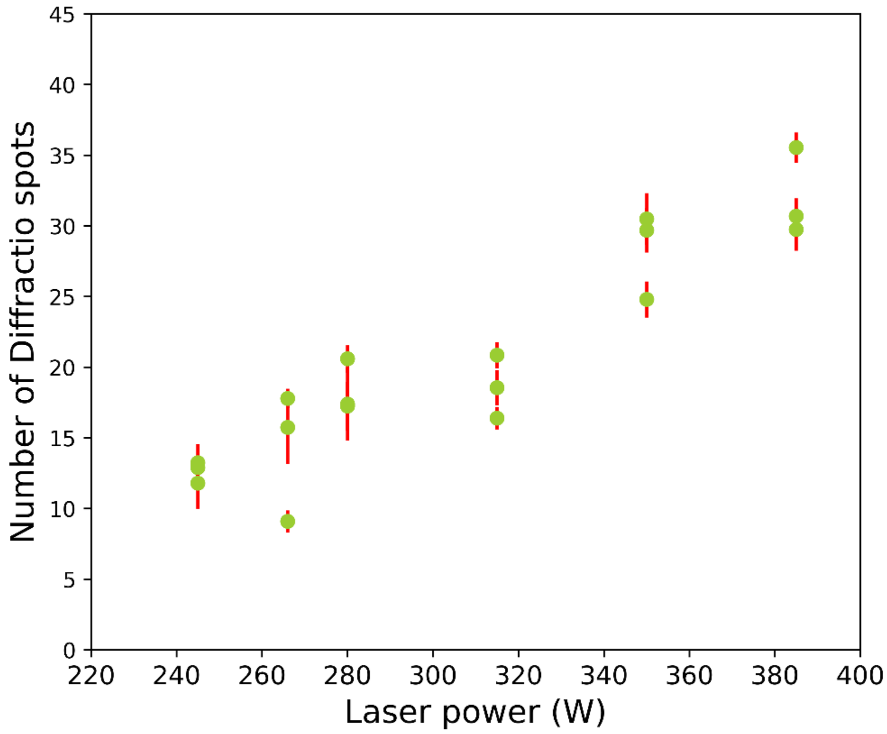

Supplementary Fig. 4. Number of diffraction spots in additive manufacturing process with 1 layer of powders under different laser powers. Error bars indicate the standard deviation coming from 20 repeatedly recorded data in the same sample.

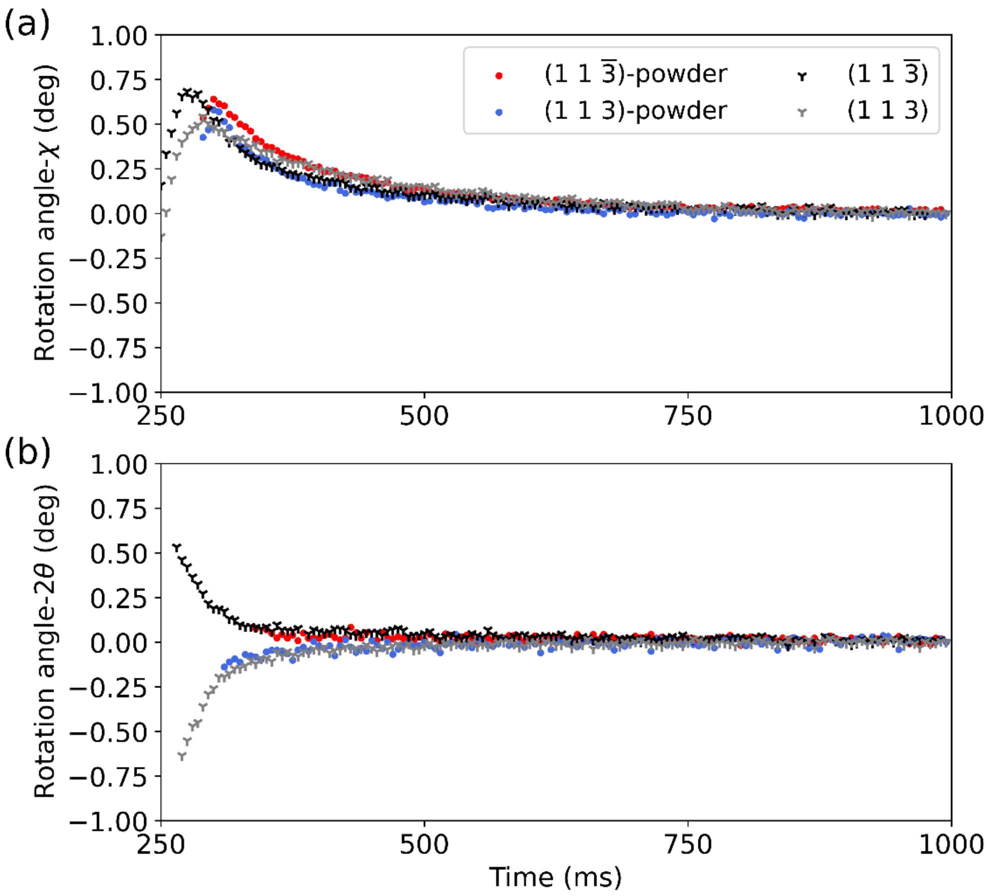

Supplementary Fig. 5. Dynamic evolution of the $\gamma\,(11\bar{3})$ and $\gamma\,(113)$ diffraction peaks during solidification of LPBF and laser remelting processes. (a-b) The rotation angle in the χ (a) and 2θ (b) directions versus time. The red and blue dots represent the $\gamma\,(11\bar{3})$ and $\gamma\,(113)$ diffraction peaks during LPBF, respectively. The black and grey symbols represent the $\gamma\,(11\bar{3})$ and $\gamma\,(113)$ diffraction peaks during laser remelting process, respectively.

The dynamic crystal rotation behaviour of the additive manufacturing process with powders are similar to those observed on a bare plate. In the comparative experiments, the X-ray was located in the interface between the powder and substrate, and both the powder and substrate probed. The evolution of the $\gamma(11\bar{3})$ and $\gamma(113)$ diffraction spots during solidification processes with and without powders is displayed in Supplementary Fig. 5. The rotation angle along the 2θ and χ directions with powders overlaps well with that of the bare plate.

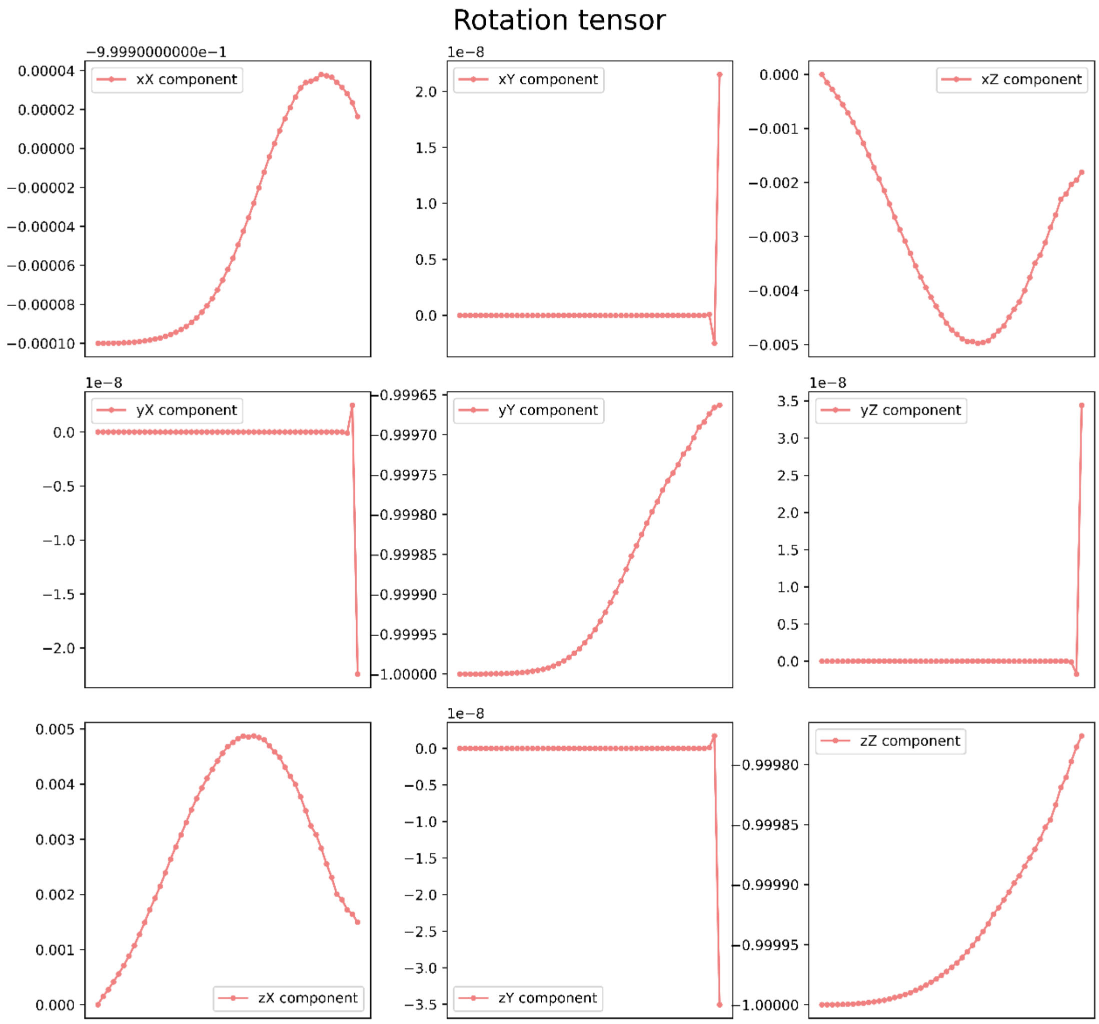

Supplementary Fig. 6. Temporal evolution of the rotation tensor during laser heating process (150 – 210 ms).

As shown in Supplementary Fig. 6, the xY, yX, yZ and zY components of rotation tensor remain close to zero throughout the entire laser heating process. This suggests that the rotation tensor **R** corresponds to a single **Y**-rotation operation, and thus, R_{xZ} equals $\sin\chi$, where χ means the **Y**-rotation angle. The positive direction of rotation is indicated by the clockwise direction.

Contributions to the diffraction peak width change (FWHM)

Energy bandwidth: The energy spectrum used in this work is generated from a superconducting wiggler, as shown in Supplementary Fig.7. The broad band "white beam" contributes little to the diffraction peak width change.

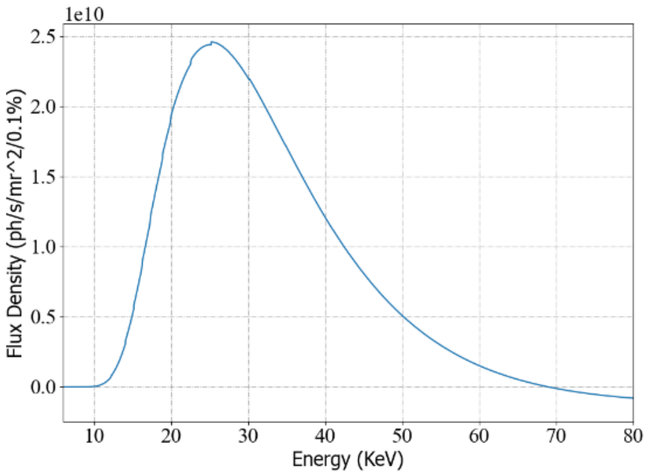

Supplementary Fig. 7. Energy spectrum of white beam X-ray generated from a superconducting wiggler

Instrumental factors: The broadening of the detector and other instruments remains constant in the experiment, so it will not affect the change of FWHM over time.

Grain size effects: According to the Scherrer formula, the smaller the grain size, the greater the broadening, and vice-versa. When the grain size reaches micron level, the peak width caused by the grain size effect becomes negligible, and other factors start to controll it. For additive manufactured materials, as shown in Fig. 1b, the solidified grain size perpendicular to the build direction (**X**- and **Y**-axis) reaches micron level, while it is bigger along the build direction (**Z**-axis). Meanwhile, due to the high cooling rate, the growth of grain is quite fast[3].According to the current temporal resolution (5ms), it is difficult to capture the process of the grain growth from nucleation to micrometer. Thus, the contribution of the grain size to the peak width can be ignored.

Strain gradient:

The evolution of the strain gradient with time during solidification is shown in Supplementary Fig. 8 where the time zero point is set to the beginning of solidification. The calculation details can be found in "Methods" section. Supplementary Fig. 8(a) shows the principal strain components at the farthest position from the center in the calculation area. As the strain calculated from the strain gradient tensor at this point is the largest due to the far distance from the center, it can reflect the evolution of the maximum strain over time. Supplementary Fig. 8(b)-(d) display the evolution of strain gradient tensor components associated with the principal strain over time. During the first 0.1s after solidification, the strain gradient is large, but rapidly decreases during 0.1 - 0.2 s. After about 0.2 s, some strain gradient tensor components gradually increased in the reverse direction, and finally, all components tended to a stable value with a length smaller than the initial value.

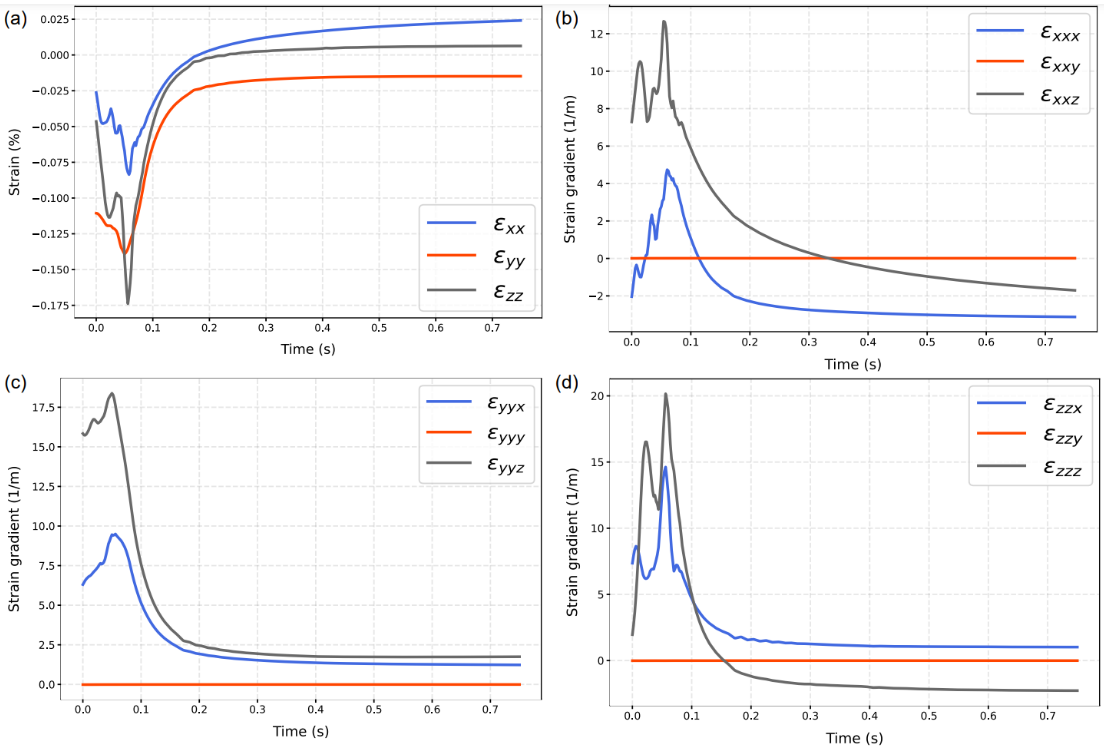

Supplementary Fig. 8. Evolution of the strain gradient with time during solidification. (a) The principal strain components at the farthest position from the center in the calculation area. (b-d) The evolution of the strain gradient tensor components associated with the principal strain with time. The time zero point is set to the beginning of solidification.

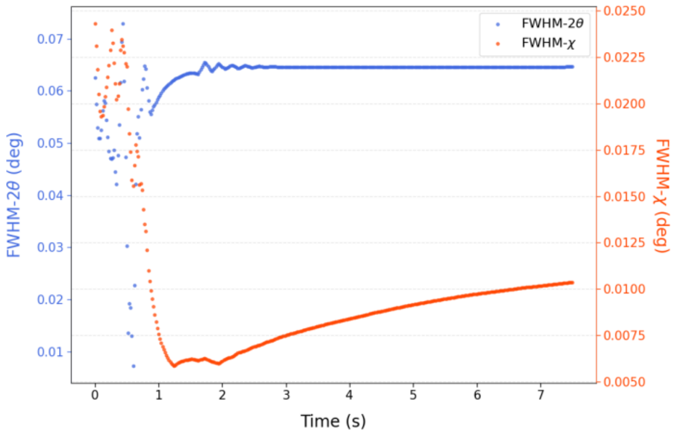

Supplementary Fig. 9. Evolution of FWHM of the γ($\bar{3}\,\bar{1}\,\bar{1}$) crystal plane caused by the strain gradient during solidification. The blue and orange dots represent FWHM along the *2θ* and *χ* directions, respectively. Other crystal planes show the similar results

For this complex strain case, we performed diffraction simulation in the calculation area based on the results presented in Supplementary Fig. 8. The simulation allowed us to track the evolution of FWHM induced by the strain gradient over time, as illustrated in Supplementary Fig. 9. No matter along the *2θ* or *χ* directions, the diffraction peak width remained within a very narrow range (*2θ* < 0.07°, *χ* < 0.03°) compared with our experimental results (Fig. 3). Therefore, the strain gradient has a negligible contribution to the observed diffraction

broadening.

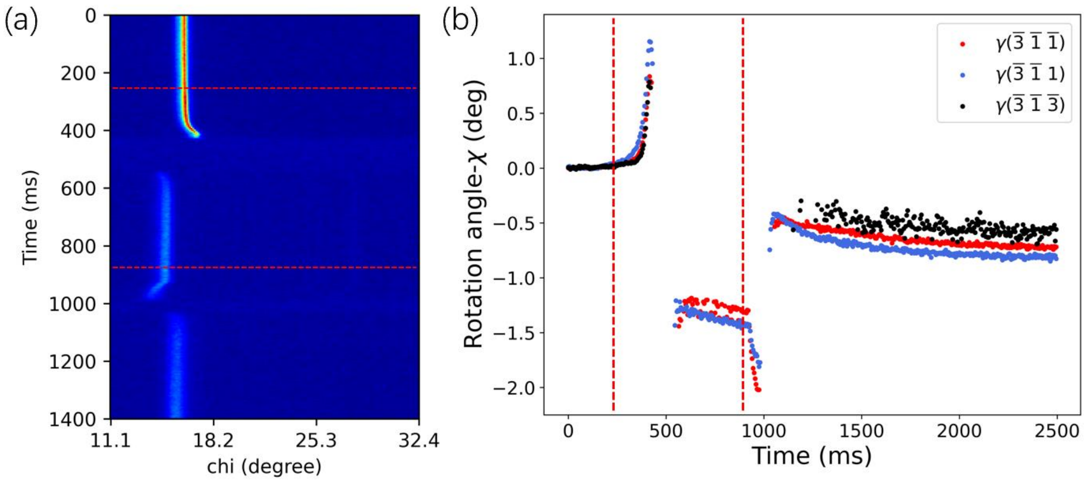

Supplementary Fig. 10. Dynamic evolution of diffraction peaks during the laser reciprocating scanning process. (a) Variation diagram of the $\gamma\ (\bar{3}\ \bar{1}\ \bar{1})$ lattice plane with time in the χ direction. (b) The rotation angle in the χ direction versus time. The red dotted lines represent the moment when the laser was turned on.

It is important to notice that the deformation gradient and thermal gradient field are dependent on the laser scanning direction. Therefore, if the laser scans in the opposite direction, the crystal rotation direction should also be opposite. Additionally, after laser reciprocating scanning, the crystal should be essentially rotated to its initial orientation due to the opposite deformation gradient and thermal gradient fields. To confirm this, we conducted an experiment with laser reciprocating scanning and performed Laue diffraction at a recording rate of 200 frames per second. The first laser scanning direction was along the -**X** direction at a laser power of 350 W, while the second scanning direction was along the **X** direction at a laser power of 300 W.

The results, displayed in Supplementary Fig. 10, reveal that the crystal rotation directions of the two processes with opposite scanning directions are opposite, and the orientation eventually returned to the initial state. This further validates our explanation that during the laser remelting process the localized heating/cooling heterogeneity-induced material deformation gradient field governs the crystal rotation. Thus, an opposite scan strategy is an optional method to maintain a single-crystal texture along the building direction.

Supplementary References

1. Huang, X. R. LauePt, a graphical-user-interface program for simulating and analyzing white-beam X-ray diffraction Laue patterns. *J Appl Crystallogr* **43**, 926–928 (2010).
2. Huang, J. W., Zhang, Y. Y., Hu, S. C., Cai, Y. & Luo, S. N. DATAD: A Python-based X-ray diffraction simulation code for arbitrary texture and arbitrary deformation. *J Appl Crystallogr* **54**, 686–696 (2021).
3. Gao, Z. & Ojo, O. A. Modeling analysis of hybrid laser-arc welding of single-crystal nickel-base superalloys. *Acta Mater* **60**, 3153–3167 (2012).
