# Supplementary material for: In situ observation of crystal rotation in Ni-based superalloy during additive manufacturing process
Source: Nat Commun. 2023 May 23;14:2961. doi: 10.1038/s41467-023-38727-8 (PMC10206100; doi:10.1038/s41467-023-38727-8)
Supplement: Supplementary file 3 — Description of Additional Supplementary Files [file 41467_2023_38727_MOESM3_ESM.pdf]

**Description of Additional Supplementary Files**

File Name: Supplementary Movie 1
Description: Evolution of diffraction patterns during laser remelting process of a single-crystal nickle-based superalloy bare substrate at laser power of 266 W and scan speed of 0.02 m $s^{-1}$.

File Name: Supplementary Movie 2
Description: Evolution of diffraction patterns during laser remelting process of a single-crystal nickle-based superalloy bare substrate at laser power of 245 W and scan speed of 0.02 m $s^{-1}$.

File Name: Supplementary Movie 3
Description: Evolution of diffraction patterns during laser remelting process of a single-crystal nickle-based superalloy bare substrate at laser power of 350 W and scan speed of 0.02 m $s^{-1}$.

File Name: Supplementary Movie 4
Description: Evolution of diffraction patterns during laser remelting process of a single-crystal nickle-based superalloy bare substrate at laser power of 385 W and scan speed of 0.02 m $s^{-1}$.

File Name: Supplementary Movie 5
Description: Evolution of diffraction patterns during laser powder bed fusion process of single-crystal nickle-based superalloy at laser power of 280 W and scan speed of 0.02 m $s^{-1}$.

File Name: Supplementary Movie 6
Description: Simulated shift of diffraction spots when the crystal rotates along the **X** axis. White dots are the simulated results.

File Name: Supplementary Movie 7
Description: Simulated shift of diffraction spots when the crystal rotates along the **Y** axis. White dots are the simulated results.

File Name: Supplementary Movie 8
Description: Simulated shift of diffraction spots when the crystal rotates along the **Z** axis. White dots are the simulated results.
